# Supplementary figures and images for: Proliferation of group II introns in the chloroplast genome of the green alga Oedocladium carolinianum (Chlorophyceae)
Source: PeerJ. 2016 Oct 25;4:e2627. doi: 10.7717/peerj.2627 (PMC5088586; doi:10.7717/peerj.2627)

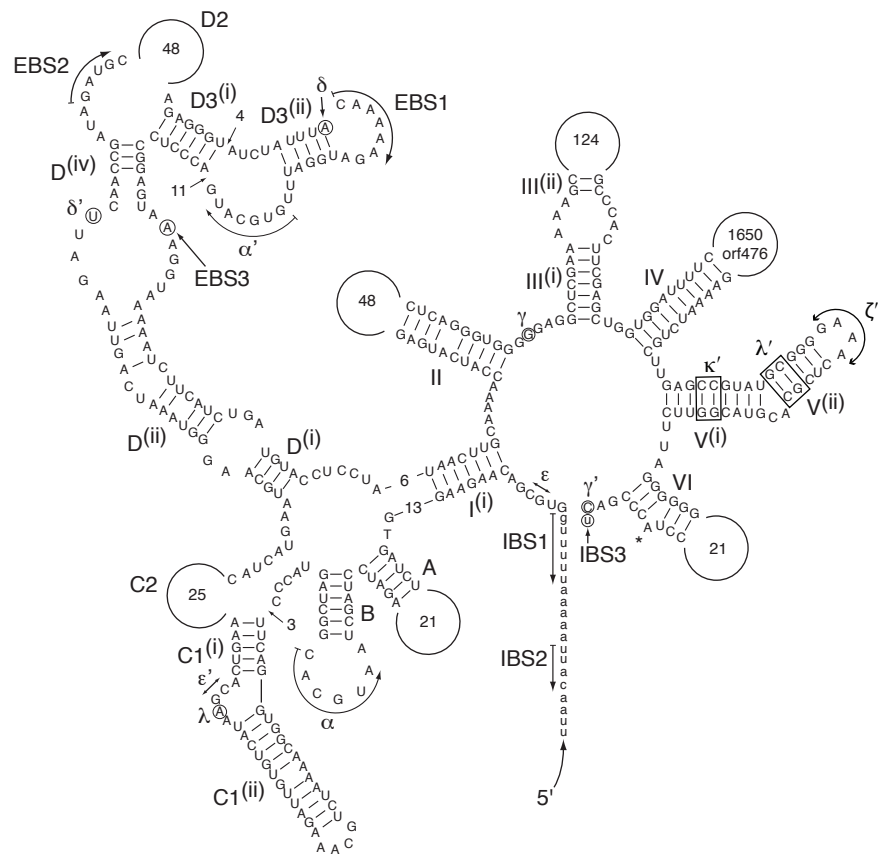

Supplement: Figure S1 — Exon sequences are shown in lowercase letters. Roman numerals specify the six major structural domains of group II introns. Blocked arrows and Greek letters denote nucleotides involved in tertiary interactions. EBS and IBS refer to exon-binding and intron-binding sites, respectively. An asterisk denotes the putative site of lariat formation. Numbers inside the loops denote the sizes of these regions. [file peerj-04-2627-s001.pdf]

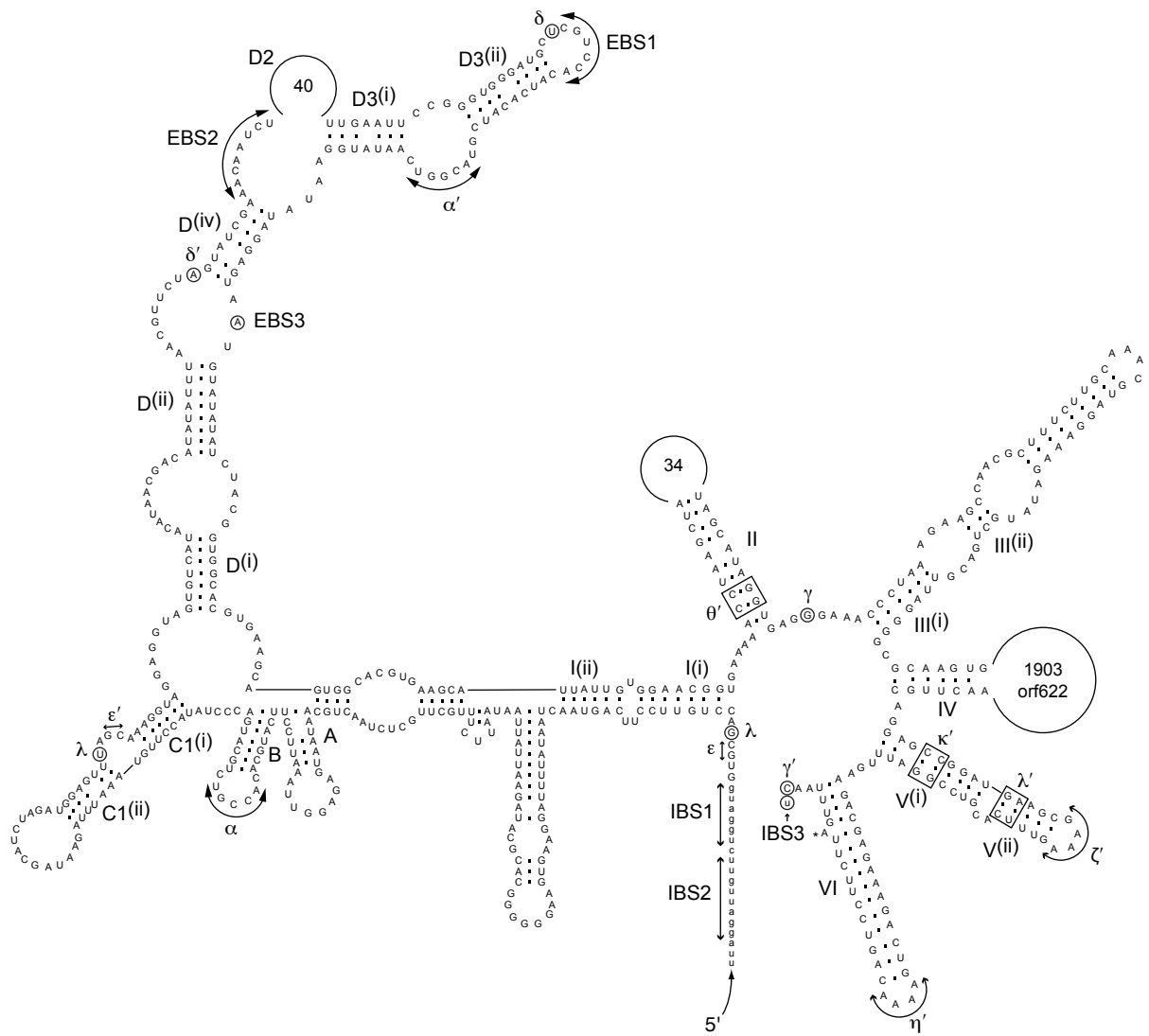

Supplement: Figure S2 — Exon sequences are shown in lowercase letters. Roman numerals specify the six major structural domains of group II introns. Blocked arrows and Greek letters denote nucleotides involved in tertiary interactions. EBS and IBS refer to exon-binding and intron-binding sites, respectively. An asterisk denotes the putative site of lariat formation. Numbers inside the loops denote the sizes of these regions. [file peerj-04-2627-s002.pdf]
